# Regulation of Mouse Retroelement MuERV-L/MERVL Expression by REX1 and Epigenetic Control of Stem Cell Potency

**DOI:** 10.3389/fonc.2014.00014

**Published:** 2014-02-06

**Authors:** Jon Schoorlemmer, Raquel Pérez-Palacios, María Climent, Diana Guallar, Pedro Muniesa

**Affiliations:** ^1^Regenerative Medicine Program, Instituto Aragonés de Ciencias de la Salud, Zaragoza, Spain; ^2^ARAID Foundation, Zaragoza, Spain; ^3^Departamento de Anatomía, Embriología y Genética Animal, Facultad de Veterinaria, Universidad de Zaragoza, Zaragoza, Spain

**Keywords:** MERVL, REX1/ZFP42, embryonic stem cells, two-cell state, developmental potential

## Abstract

About half of the mammalian genome is occupied by DNA sequences that originate from transposable elements. Retrotransposons can modulate gene expression in different ways and, particularly retrotransposon-derived long terminal repeats, profoundly shape expression of both surrounding and distant genomic *loci*. This is especially important in pre-implantation development, during which extensive reprograming of the genome takes place and cells pass through totipotent and pluripotent states. At this stage, the main mechanism responsible for retrotransposon silencing, i.e., DNA methylation, is inoperative. A particular retrotransposon called *muERV-L/MERVL* is expressed during pre-implantation stages and contributes to the plasticity of mouse embryonic stem cells. This review will focus on the role of MERVL-derived sequences as controlling elements of gene expression specific for pre-implantation development, two-cell stage-specific gene expression, and stem cell pluripotency, the epigenetic mechanisms that control their expression, and the contributions of the pluripotency marker REX1 and the related Yin Yang 1 family of transcription factors to this regulation process.

## Introduction

Transposable elements (TEs) are DNA sequences with the ability to move from one chromosomal location to another. They were discovered by McClintock (1), who was awarded the 1983 Nobel Prize in Physiology or Medicine for this work. To explain color patterns in maize kernels, she postulated that gene expression might be regulated by “controlling elements” that could jump around the genome [reviewed by Fedoroff (2)]. Initial drafts of both human and mouse genomes indicated that about half of the mammalian genomes are occupied by DNA sequences that originate from TE (3). Posterior estimates are even higher up to two-thirds (4). Among the TEs figure the endogenous retroviral elements (ERVs), which make up about 10% of mammalian genomes. As opposed to exogenous retrovirus, ERVs are an integral component of the genome in all cells of an organism. ERVs are the remnants of ancient retroviral infections of the germline that have produced stable integrations in the genome, which are therefore passed on to the offspring in a Mendelian fashion (5, 6). Expression of ERV is usually repressed by mechanisms dependent on DNA methylation (7), and deregulation of human ERV (HERV) contributes to disease (8). In contrast to other mammals like mouse and cat, there exists no definitive proof for the presence of infectious HERV particles, although ERV might be mobile even in human (9). Therefore, HERV might contribute to tumor development as retroviruses do (8), through HERV-encoded transcripts, or by regulating expression of genes nearby or even at a distance. Indeed, the expression of HERVs has been linked to a variety of tumors (8, 10), although HERV has not presently been identified as an etiological factor in tumor development.

Pluripotent self-renewing embryonic stem (ES) cells can be derived from the inner cell mass (ICM) of the pre-implantation blastocyst. Cells from earlier embryos (zygotes until eight-cell embryos) differentiate into both fetal and placental cell types. This ability is called totipotency as opposed to the more restricted differentiation potential of pluripotent ICM cells, which only contribute to embryonic tissue (11, 12). ES cells in culture are heterogeneous (13, 14), and several of their characteristics can best be understood in the context of successive changes occurring during pre-implantation development, as the cells transition between states that resemble cellular identities at distinct stages of pre-implantation development. Mouse ES cells display special epigenetic features such as a loose chromatin structure and altered DNA methylation, which underlie their cell type-specific properties (15). Similar to the pre-implantation embryo, mouse ES cells express several kinds of ERV, in line with the absence of DNA methylation. Among the ERV expressed is *MERVL*, whose expression actually peaks in two-cell stage embryos (16). Importantly, *MERVL* expression in mouse ES cells is restricted to a subpopulation of the cells with special characteristics as defined by gene expression and differentiation potential normally found in cells of two-cell embryos (17). Degenerated cis-acting elements derived from *MERVL* elements are also essential to pluripotency-related phenomena, i.e., re-activation of the second X-chromosome (18, 19), and we speculate on its role in developmental potency and differentiation.

We will review how *MERVL* sequences drive gene expression restricted to different states of developmental potency in mouse stem cells. These contributions depend on epigenetic regulation mechanisms exerted by protein complexes that modify or read histone modifications, particularly Polycomb complexes and demethylase complexes. REX1 is a pluripotency-associated nuclear protein that binds *MERVL* elements *in vivo*, and a contribution of REX1 to silencing of *MERVL* has been demonstrated (20). REX1 reunites additional characteristics consistent with a role in orchestrating how regulatory complexes interact with and shape *MERVL*-dependent transcription. We present a hypothetical model incorporating this potential role for REX1. As studies to determine the role of *HERV* -driven processes for human ES cells (HESCs) are ongoing, we will briefly review differences with the mouse and explain outstanding questions.

Apart from pluripotent cells, several TEs including ERV are active and expressed in the germline, in pre-implantation embryos, and in the placenta (21, 22). These are exactly the tissues relatively devoid of DNA methylation (23), similar to the epigenetic chromatin state in many tumors (24), which is also characterized by widespread DNA hypomethylation. At present, there is little information on the potential activity of other repressing mechanisms toward TE silencing, apart from the main methylation-dependent mechanism (7). However, we suggest that the epigenetic mechanism operative in ES cells described here may carry out such functions. We hope that a better understanding of the DNA-binding factors and their interactions with chromatin modifying regulators may advance future understanding of the relationship between HERV regulation and tumor prevention. We therefore point out the activity of similar mechanisms in human cells and consider their potential relevance for tumor formation and/or progression.

## Transposable Elements and Endogenous Retroviral Elements

Transposable elements exist as either DNA transposons that directly jump from one location to another or as so-called retrotransposons that use an RNA intermediate (which in turn is retro-transcribed into DNA before reinsertion into the genome). Retrotransposons can be further divided into long terminal repeat (LTR)-containing TEs (LTR retrotransposons and ERV) and non-LTR retrotransposons LINE and SINE (long and short interspersed nuclear elements).

Long terminal repeats are generated during the reverse transcription step. LTRs recruit the cellular transcription factors (TFs) in charge of proviral transcription and produce the 5′ and 3′ ends of the transcripts (25). In mammals all LTR transposons are related to ERV, which are considered (defective) descendents of ancient retroviral infections of the germline.

Animal retroviral diseases (i.e., Jaagsiekte in sheep) were already described at the turn of the nineteenth century, and particles derived from ERV were identified in the late 1960s in birds and mice. Also, in different mammalian species abundant expression of ERV in placenta and trophoblastic cells had been known since the 1970s (26–28). Reverse transcriptase (RT) assays and electron microscopy were used to identify additional ERV particles, and ERV-reactive antibodies were searched for in sera and other body fluids. The understanding of ERV was limited however, as neither the integration of RNA in the genome, nor non-Mendelian genetics were widely accepted concepts at the time.

Much was learned about ERV in the course of studies on tumor-producing virus in chicken, especially avian endogenous leukosis virus (ALV) and the closely related Rous sarcoma virus (RSV), using a combination of virological and immunological methods common at the time or developed for this purpose [reviewed by Weiss (29)]. Neutralizing sera against envelope proteins were available and serological tests were developed for “group-specific antigen” (GAG) common to serotypes. This allowed the detection of GAG protein in non-infected animals, suggesting the endogenous presence of this viral protein. Mendelian transmission of virally derived characteristics was established in appropriate crosses (i.e., between Gag-positive and negative inbred lines). Furthermore, the use of nucleic acid hybridization allowed for a positive identification of endogenous copies very similar to virus-derived RNA. HERV was first discovered in normal brain tissue (30), and similar to other mammalian species the human placenta was shown to be permissive to the expression of HERV.

As repeated DNA elements, ERVs were included in the category of “junk” DNA and long considered not relevant. More detailed analysis has further been hampered by cross-hybridization among related elements, and both element-specific probes and amplification assays have been scarce for a long time. Extensive sequencing projects that have delivered complete mouse and human genomes, have finally provided tools and information to appreciate the extension and importance to the genome of TE and ERV in particular (21).

**Figure 1:**
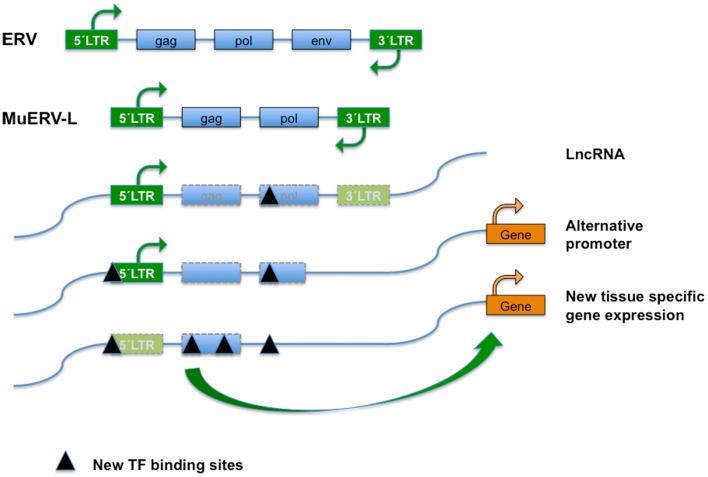
**Endogenous retroviral elements (ERVs) result from the occasional infection of a germline cell by a retrovirus, whose (potentially) defective offspring is transmitted further as a provirus in a Mendelian fashion**. ERVs typically encode the canonical retroviral proteins GAG (group-specific antigens; capsid and matrix protein), POL (RT, reverse transcriptase; IN, integrase), and ENV (envelope). The ERVs are divided into three classes based on the sequence conservation with active retrovirus; class I (eMLVs), class II (musD/ETn/IAPs), and class III (ERV-L). ERVs are flanked by LTRs (green boxes) of 300–1,200 nucleotides as a result of the retrotransposition event. Class III ERVs make up almost 5% of the mouse genome and consists of muERV-L/MERVL elements (depicted) and the non-autonomous mammalian apparent LTR retrotransposons (MaLRs). The muERV-L family members have *Gag* and *Pol* genes but no detectable *Env*, a *dUTPase* gene (not shown). For the lack of ENV they are defective for cell to cell transmission. In addition to muERV-L, the mouse genome contains many thousands of ERV-L derived LTR elements termed MT2-Mm. LTRs are bound by transcription factors (TF) that drive transcription. ERV elements may also bind TF in the body of their genes, probably influencing chromatin dynamics or surveillance. After initial insertion (and transposition), ERVs over time may accumulate mutations and have contributed significantly to the generation of new transcription factor binding sites and hence impact on gene expression, genome function, and evolution. Transposon-derived promoters drive expression of alternative transcripts, including many of the LncRNAs detected by the ENCODE project, orchestrate stage, and tissue-dependent transcription, or serve as alternate promoters.

As opposed to exogenous retrovirus, ERVs are an integral component of the genome in all cells of an organism (5, 6). Posterior to integration, re-activation, amplification, and transposition events have produced defective proviral derivatives of variable integrity dispersed throughout the genome. In the most extensive cases of genetic lesion/degeneration only isolated LTRs are left as molecular fossils of previous integrations. Autonomous ERV encode the canonical retroviral GAG (capsid and matrix protein), POL (RT; IN, integrase), and ENV (envelope proteins) (Figure 1). Although over the years ERVs have mutated and accumulated defects in the coding regions of some or all of their genes, some still have open reading frames (ORFs) and thus direct the expression of (a subset of) these proteins. Dependent on the amount of mutations acquired, autonomous elements can still recruit the necessary cellular machinery to produce infectious particles, while non-autonomous variants rely on related elements to do so. Although TEs are classified based on sequence similarity, each group consists of a range of similar but clearly distinguishable elements. Furthermore, within each group copy numbers and autonomy status differs between individual elements (5).

Based on the sequence of their RT genes and their relationship to described *genera* of exogenous retroviruses, ERVs are divided into three families or classes (31): ERVI/class I, ERV-K/class II (IAPs and MusD/ETn), and class III (ERV-L/MaLR). Class I ERVs comprise <1% of the mouse genome and this class is typified by the well-studied type-C murine leukemia viruses (MuLV), and also includes eMLVs, VL30, and MuRRS elements. Mouse mammary tumor virus (MMTV) is typical of the class II ERVs, which also includes IAP, musD, and deleted musD variants called ETn elements. MusD elements contain *Gag-Pro-Pol* genes, but lack an *Env* gene and vary from 5 to 7 kb in length. ETns are flanked by LTRs but mainly contain sequences of unknown function.

The Class III ERV in the mouse consists of two types of retrotransposon elements: murine ERV-L elements (muERV-L/MERVL) and the mammalian apparent LTR retrotransposons or MaLRs. After its initial identification in humans (32) the ERV-L family turned up in all placental mammals. The ERV-L family members are about 6.5 kbp in length and carry *Gag* and *Pol* but no *Env* genes, and carry an extra *dUTPase* gene (Figure 1). MERVL/MuERV-L (mouse ERV with a leucine tRNA primer binding site), is present in over 650 full-length copies in the C57BL/6 genome.

In addition, the C57BL/6 genome carries 37,000–38,000 copies of *MERVL*-derived LTRs called MT2-Mm. MT2 (MERVL) and MaLR LTRs share about 50% homology at the DNA level. MaLRs are very common, non-autonomous LTR elements that occupy 4.8% of the mouse genome. Among the MaLR retrotransposons three subfamilies are discerned, termed MT (about 2 kb), ORR1 (2.5 kb), and MLT. The non-autonomous but active MaLRs are all internally deleted, containing only non-coding repetitive DNA flanked by LTRs.

Elements of active TE/ERV families in the mouse (i.e., IAP and ETn in the mouse) display high levels of polymorphism, contributing to genetic variability within species. Elements detected in one strain (C57BL/6J) are absent in other three strains investigated (60 and 25% of IAP and of ETn/MusD elements, respectively), and differences in expression levels or the generation of alternative transcripts of ERV elements was also observed (33).

The activity of TEs in general and ERV in particular may contribute to pathogenic processes in different ways (1). Stress signals resulting from injury, infection, or inflammation may relieve repression of ERVs, provoking the expression and assembly of infectious virions. Independent of the pathogenicity of the resulting virions, reintegration may damage the genome (2). De-repressed ERVs may direct the production of retroviral proteins that either contribute to cell fusion (34) or display characteristics as superantigens causing inflammation (3, 35). Altered gene activity of ERV *loci* can affect the expression of neighboring cellular genes (25, 36).

To avoid deleterious effects of TEs/ERVs activation of any kind, mammalian cells have acquired a multitude of defense responses. They act at different stages of the TEs life cycle starting with proviral transcription and processing of resulting RNAs. Other mechanisms interfere with various steps required for productive infection and integration of retrovirus into the genome: release of particles, receptor binding, uncoating, deamination (APOBEC enzymes), and intracellular trafficking (31). In the mouse germline, transcriptional repression of retrotransposons is regulated through DNA methylation of their regulatory LTR regions (37). This process is dependent on co-operating small RNAs (piRNAs) and PIWI-proteins in a mechanism unique to the germline in mammals (38). This review will focus on the role of transposons as controlling elements of pre-implantation development and stem cell pluripotency at the level of transcriptional regulation. As there is no evidence that the PIWI pathway is relevant for these tissues, it will not be further discussed here.

## The Contribution of TE to the Evolution of Regulatory DNA

Endogenous retroviral elements largely lack transcriptional activity in differentiated cells and tissues as a result of DNA methylation-dependent silencing (7). However, expression of ERV families in different species is elevated in the germ cells, in pre-implantation embryos and also in the placenta (39–42).

Retrotransposons modulate gene expression in these tissues in different ways [reviewed in Ref. (36), see also Figure 1]. Transcription of retrotransposons may serve as a source of small RNAs that interfere with gene transcription, or produce long non-coding RNAs (lncRNAs). A substantial portion of total lncRNA sequence across species (~30% in human) is TE-derived. Conversely, TEs contribute to a full two-thirds of mature lncRNA transcripts, as relative to their absence from protein-coding transcripts (43). TEs and particularly retrotransposon-derived LTRs profoundly shape expression of surrounding genomic *loci* through long distance enhancer or repressor activities, which may favor the use of alternative promoters, exons/splice sites, or polyadenylation sites (25, 36, 44).

It has been argued that the accumulation of TE in the genome has provided an abundant source of non-functional DNA with progressive mutations for evolution to act upon. Indeed (degenerated) LTR from ERV have been frequently recruited as binding sites for TFs (45). A well-studied example is the expansion of binding sites for the TF CTCF (CCCTC-binding factor), a DNA-binding protein that insulates transcriptional and chromatin domains (46). Mouse ES cells have thousands of CTCF-binding sites that are not conserved in the human genome, as they are found in B2 retrotransposons (47), a rodent-specific SINE, which originates from accidental retrotransposition of various polymerase III transcripts (31). Species-specific activation of retroelements has generated novel species-restricted CTCF-binding sites with active chromatin insulator function also in other species including rodents, dogs, and opossum (48).

Similarly, specific families of TE are enriched for TF binding sites, for example Class II elements for SOX2 and POU5F1 binding (47). Similar to the extension of CTCF-binding sites, regulatory elements carried on by transposons contribute to differences in gene expression patterns between different mammalian species (49). In line with remarkably restricted and regulated expression patterns, retrotransposon-directed transcription contributes to gene regulation in the endometrium (50), and during pre-implantation development (40). Transposon-derived promoters synchronize stage-specific gene expression in two-cell embryos (36, 51) and ERV-derived cis elements have been incorporated in regulatory networks in pluripotent stem cells (see below).

## Pre-Implantation Development

**Figure 2:**
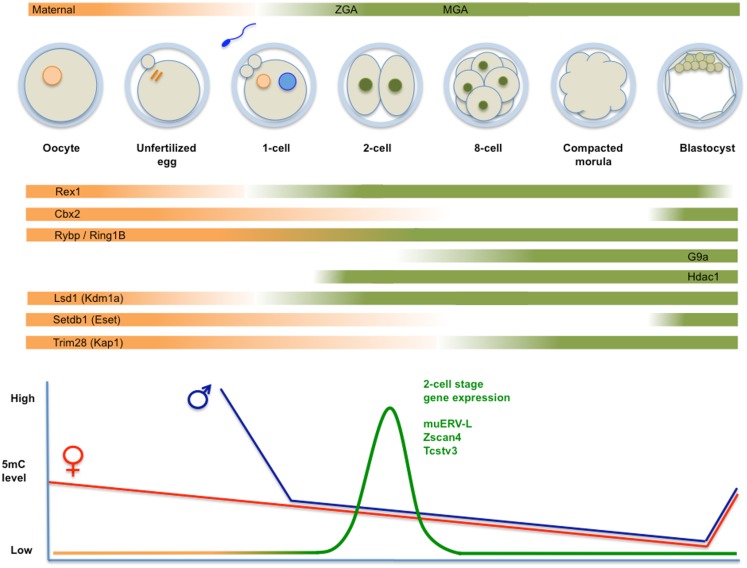
**Top panel: schematic representation of fertilization, formation of the zygote, and cleavage divisions, followed by the successive generation of morula and blastocyst**. Transcripts derive from maternal deposits, or correspond to the two transient waves of *de novo* transcription: zygotic gene activation (late zygote-two-cell) or ZGA, and mid pre-implantation gene activation (MGA). The temporal changes in expression levels of important chromatin modifiers are indicated. Bottom panel: time course of genome-wide (de)methylation. By the time of implantation, DNA methylation is re-established and TE are rapidly silenced. Demethylation is not uniform, i.e., certain TE (i.e., IAP) and imprinted *loci* are protected from demethylation. The diagram also shows a peak of unique expression of muERV-L, Zscan4, Tcstv3, and others in two-cell embryos.

After fertilization, the mammalian zygote develops into a multi-cellular blastocyst, which implants in the uterus for further development. Already at the first stage, genome-wide reorganization takes place to ensure that after protamine/histone exchange and demethylation of the male genome and the initiation of replication of haploid genomes, fusion of pronuclei takes place. Large-scale chromatin reorganization includes but is not limited to epigenetic modifications that are intertwined with zygotic gene activation (ZGA). Methylated cytosines are removed from both parental genomes in early cleavage embryos (23). While the maternal genome is passively and progressively demethylated during cleavage divisions (Figure 2), the paternal genome is actively demethylated in the zygote, at least in the mouse (52). During these early stages, a distinct chromatin structure and nuclear architecture are in place concomitant with the absence of structural restrictions to transcription.

Several excellent reviews detail the progress of cell division followed by cellular differentiation steps during pre-implantation development and the accompanying changes in chromatin states (15, 53, 54) (see also the legend of Figure 2). The diploid zygotic genome results from the gradual (55) fusion of paternal and maternal genomes into a totipotent embryo, referring to the ability to give rise to both embryonic and placental tissues. This property is maintained during cleavage divisions up to the eight-cell stage. Afterward, cell specification is initiated, concomitant with a loss of totipotency, which is definitive at the 32-cell stage.

A next major event is compaction of the loosely connected cells into a more tightly associated structure, followed by the successive generation of morula and blastocyst. Simultaneous with compaction cell polarization takes place, which initiates a first cell differentiation, separating cells in the ICM from the surrounding Trophectoderm. Posteriorly, in the late blastocyst cells in the ICM separate into epiblast (or primitive ectoderm) and primitive endoderm (or hypoblast) in a stochastic process (56). As a result of these two successive differentiation steps, the late blastocyst contains three separated cell types or lineages: the trophectoderm, the primitive endoderm, and the epiblast. While the trophectoderm and the primitive endoderm contribute to extraembryonic tissues, the epiblast gives rise to all cell types of the developing embryo proper. Once these lineages have been specified in the early embryo, they are separated and become committed to a particular fate.

Formation of the zygote and posterior cleavage divisions are associated with specific mechanisms of regulation as a result of specific epigenetic features and the spatial organization of the newly formed diploid genome (53, 57). These include but are not limited to differential methylation of histones and DNA, as well as active DNA demethylation (see below and Figure 2). This period is associated with specific patterns of gene expression (40, 58, 59) that reconcile these mechanistic features with developmental requirements.

Among the earliest expressed genes are several MaLR and muERV-L family transposons (the latter collectively referred to as *MERVL*), and chimeric transcripts that initiate on them. In the mature oocyte/zygote, MaLR-derived sequences are overrepresented in transcripts (17, 40, 60). In two-cell embryos, muERV-L itself displays a transient increased expression (16) and *MERVL* sequences are incorporated into epsilon virus-like particles observed in the early mouse embryo (61). In addition to *MERVL* itself, over 50 transcripts have been identified that are expressed in two-cell stage embryos and are directly linked to *MERVL* elements (17). We will refer to these genes as LTR-linked 2C genes.

By contrast, class II IAP and musD/ETn elements are expressed from the blastocyst stage onward (62). ERV-driven gene expression is important at these stages, as at least in the case of LINE1 elements (L1Md_T class) (63) as well as muERV-L elements (64), cleavage division is impaired upon targeted depletion by antisense oligonucleotides.

## Cellular Heterogeneity and Naive Pluripotency

Mouse ES cells can be derived from the ICM of blastocyst embryos (12), a stage posterior to ICM and trophectoderm lineage separation. ES cells share several characteristics with cells in the ICM of the blastocyst, and can differentiate *in vitro* into all the cell types of a *fetus* (including the germline). ES cells are considered pluripotent because in chimeric embryos they contribute to each of the three germ layers of the embryo proper (11). Only rarely do they contribute to the extraembryonic tissues in the placenta, providing a distinction with totipotent cells in the two-cell embryo. ES cells can be maintained in culture for an apparent indiscriminate number of cell divisions (a property called self-renewal).

Maintenance of ES cells *in vitro* depends on extracellular signaling by LIF and BMP4. Signals converge on the OCT4, NANOG, and SOX2 TFs, which mutually stimulate each other’s expression, and repress genes either promoting or associated with cell differentiation. Together these core pluripotency factors form a co-operating network of TFs, whose activity also relies on the recruitment of epigenetic regulators (13, 65, 66). Contrasting with the uniform expression of OCT4 and SOX2 in all pluripotent cells of mouse ES cell cultures, heterogeneous expression has been observed for several genes including *Zfp42/Rex1* (67), *Nanog* (68, 69), *Stella* (*Dppa3/Pgc7*) (70), *Esrrb, Pecam1* (71, 72), and *Zscan4* (72, 73). The exact relationship between expression of these various markers is unknown. At least in the case of *Stella, Pecam1*, and *Rex1*, expression seems to mark the same cell population (70).

Interestingly, after selection for high expression of such factors, often a heterogeneous population is reconstituted spontaneously (67, 70) and cells negative for markers such as NANOG, REX1, or STELLA can regain expression for each of these factors. Therefore, cells shuttle between high and low expressing states and the resulting heterogeneity reflects the presence of subpopulations of cells, as opposed to an irreversible differentiation status. The differences in gene expression levels between subpopulations may also define distinct self-renewal and differentiation properties, which together define developmental potency. High expression of those genes correlates with self-renewal *in vitro* and enhanced contribution to chimeric embryos. Cells with low expression show increased (but reversible) expression of differentiation markers (67, 70, 74), exhibit enhanced sensitivity to differentiation-inducing conditions *in vitro* (70) and show altered contribution to chimeric embryos (67).

Different models have been proposed to describe this cellular heterogeneity. According to a first model, cells transition between metastable states referred to as “naive” and “primed.” The transition between different states is determined by differentiation-inducing ERK signaling, in such a way that inhibition of ERK signaling drives cells into a more “naive” homogeneous pluripotent state (75). In addition to compacted morphology, demethylation of the *Oct4* promoter region, silencing of retroviral vectors, two active X-chromosomes in female cells, LIF dependency and competency for germline contributions (75), this “naïve” state or ground state is characterized by homogenous expression of *Nanog* and *Rex1* (76).

## *MERVL-*Driven Transcription is Specific for the Two-Cell Stage

A distinct level of heterogeneity within ES cell cultures is related to the sporadic and transient transition of mouse ES cells into more totipotent identities. A subset of cells in a pluripotent cell culture can be separated from the other cells based on the expression of a red fluorescent protein driven by the regulatory sequences from a *MERVL*-derived LTR element (17). As differences in regulation of *MERVL*-derived LTRs have already been described (77), it remains to be established whether expression of all *MERVL* elements is equally exclusive. This particular *MERVL* element directs gene expression exclusively in two-cell embryos and in a limited number of cells (in the order of 1%) within ES cell cultures. These cells (referred to as “2C-like”) express transcripts (see Figure 2) that can be ascribed to three (overlapping) groups: (1) the *MERVL* family of ERV (but not the vast majority of other retrotransposons) and the GAG protein it encodes; (2) their corresponding LTR promoters and chimeric transcripts with junctions to *MERVL* elements; (3) a group of genes previously shown to be restricted to the two to four-cell stage of development (see Figure 1), including *Zscan4, Tcstv1/3, Eif1a, Gm4340/Thoc4, Tdpoz1–5*, and *Zfp352* (73, 78). Over 500 genes transcribed in 2C cells are also active at the two-cell stage in embryos, including 52 genes that generated chimeric transcripts linked to *MERVL* elements (17).

Surprisingly, similar to the absence of pluripotency-associated TFs OCT4, SOX2, and NANOG from two-cell mouse embryos, 2C-like cells do not express these markers at the protein level (17). In accordance with its gene expression pattern specific for two-cell stage embryos, the developmental potential of 2C-like cells is not restricted to embryonic cell types. In chimeric embryos, 2C-like cells contribute to the epiblast as expected, but also to extraembryonic tissues such as trophectoderm, the yolk sac, and placenta (17). Hence, muERV-L regulatory sequences are necessary and sufficient to drive expression in rare ES cells that express *MERVL*, and display enhanced developmental potential.

## Chromatin Structure: DNA and Histone Modifications

As opposed to genetic alterations to the genome, epigenetic mechanisms are those mechanisms that control gene expression without changing the underlying DNA sequence. Such mechanisms involve remodeling of the chromatin structure as a result of the formation of functional or 3D domains, or posttranscriptional processing in the form of DNA (de)methylation and histone modifications. A score of non-coding RNAs including microRNA, lncRNA, and piRNA also contribute to epigenetic regulation. Modifying enzymes such as DNA (DNMT) and histone methyltransferases (HMTs) are recruited by DNA-binding factors or through affinity for relevant modifications in chromatin (79). Combined, these mechanisms impact on the activity of DNA polymerase and configure the transcriptional landscape of a given genetic *locus* in a cell type-specific context.

A first major epigenetic pathway is the presence of methylated cytosine (normally in CpG nucleotides) in DNA (80). Methylation at gene promoters usually correlates with repression. DNA methylation is reversible, either by deamination and base excision repair or by TET family proteins that catalyze the conversion of 5-methylcytosine to 5-hydroxymethylcytosine, which in subsequent steps can be removed from the DNA strand (81, 82). It follows that the default role of TET1 is related to transcriptional activity (83).

A second silencing mechanism alters chromatin structure through modifications of histone tails, which are laid down by specialized enzymes that form part of histone modification complexes such as HMTs, histone deacetylases (HDACs), or lysine-specific demethylases (KDMs). The modifier activities can either stimulate or repress gene expression, depending on the affected histone residue and the overall epigenetic landscape of the *locus*. Notwithstanding, specific modifications are generally associated with a defined activity status. The trimethylation of histone 3 at lysine 4 (H3K4) is associated with active *loci*. Well-known modifications associated with repression are the trimethylation of histone 3 at K27 (involved in Polycomb-mediated repression, see below) and methylation of histone 3 at lysine 9 (H3K9), imparted by SETDB1/ESET, G9a, and the KMT1A and KMT1B products of the *Suv39h1/2* genes (84, 85).

HDAC1 belongs to the Class I HDAC, nuclear proteins with activity toward core histones (86). HDACs provide the catalytic entity for different multisubunit complexes (including SIN3A, NuRD, CoREST, and NODE). These complexes usually contribute to transcription silencing in cooperation with other chromatin modifiers, such as KDMs. A well-studied KDM is LSD1/KDM1A, which favorite substrate is mono- or di-methyl K4 in H3 (87). The activity of LSD1 can either stimulate or repress the *locus* affected. LSD1-mediated demethylation of H3K9 facilitates activation by androgen and estrogen receptors (88). By contrast, LSD1 represses as a subunit of HDAC1/2-containing CoREST complexes (89).

The interplay between the roles of DNA and histone modifying enzymes is an area of active research. Epigenetic marks and regulators are generally correlated with activity states, but do not determine them. As a generic model, *loci* are marked in a mutually exclusive way with H3K4 (active), as opposed to DNA methylation (inactive). Moreover, several instances have been described in which H3K9 trimethylation (H3K9me3)-associated silencing of Class II ERVs in ES cells, is required for posterior DNA methylation (90, 91).

## Regulation of ERV by Methylation

DNA methylation is widely recognized as an important epigenetic mark in silencing of TE and ERV (7) in line with a heavy presence of methylated CpG within repeated DNA (38). In mice, deficient in genes encoding proteins involved directly or indirectly in DNA methylation, particular TE/ERVs are de-repressed, usually leading to reduced viability and fertility (92). It has become clear however, that this mechanism does not explain the dynamics of expression/silencing in undermethylated tissues such as germ cells, pre-implantation embryos, and stem cells. As an example, although differential methylation during pre-implantation development coincides with expression of LINE1 (93) and IAP elements (94), as well as ICR regions in imprinted genes [reviewed in Ref. (95)], transient expression of muERV-L in two-cell embryos is not related to DNA methylation levels.

During mammalian pre-implantation development, DNA methylation is highly dynamic (23, 83). Until recently, it was believed that 5-methylcytosine is removed from the paternal genome immediately upon fertilization, while this occurs more slowly and passively afterward on the maternal genome, reaching a minimum at the blastocyst stage [Ref. (23), see Figure 2]. A more recent genome-wide analysis (96) has revealed that general hypermethylation of specific families of LINE1 and ERV retroelements in the sperm is rapidly removed in the zygote. Generally, retrotransposons maintain this low level of 5-methylcytosine up to the ICM stage, and do not increase to somatic levels until later (E6.5/7.5). Thus, repeat elements are undermethylated during pre-implantation stages. As an exception to this rule, high methylation levels of IAPs are retained throughout cleavage divisions (94, 96–98).

While enzymatic activity of DNMT1 and DNMT3A/B is essential for mouse embryonic development, ES cells deficient for all three genes (TKO cells) mostly retain characteristics of undifferentiated ES cells (99). Surprisingly, expression of ERV is hardly affected in TKO cells (100). While a few Class I and II ERVs depend on DNMT-mediated repression, ERVIII is totally unresponsive to TKO (100, 101).

## Silencing of *MERVL* by RYBP and Associated Polycomb Complexes

**Figure 3:**
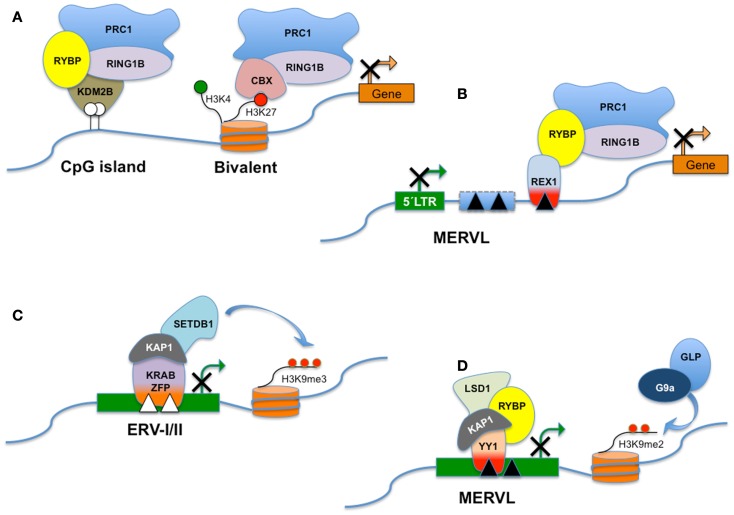
**RYBP and distinct PRC1-like complexes**. Polycomb Group genes (PcG) assemble into multiprotein complexes and, depending on the subunit composition, the complexes are recruited to different types of target sites. PRC1 (Polycomb repressive complex 1) is a canonical complex composed of either RING1A/or RING1B, MPH1/2/3, CBX2/7/8, and one of the Polycomb group RING fingers proteins (PCGF2–5) and is referred to here as CBX-PRC1 **(A)**. CBX-PRC1 mediates H3K27me3-dependent silencing of bivalent promoters. PcG-regulated promoters devoid of H3K27me3 recruit a RYBP-containing variant complex termed RYBP-PRC1, which appears similar to a KDM2B-containing complex that recognizes non-methylated CpG islands **(A)**. For clarity, only the subunits that differ between CBX-PRC1 and RYBP-PRC1 are shown, in addition to RING1B. RYBP silences the expression of transposable elements, including MERVL. RYBP can interact with both REX1 and/or RING1B. In this hypothetical model, REX1 might tether RYBP and the polycomb silencing complex to specific binding sites in MERVL or MERVL-derived control elements **(B)**. Repression of class I and class II ERVs **(C)** is dependent on SETDB1 histone H3 lysine 9 trimethylase activity (H3K9me3). SETDB1 is believed to be recruited to its ERV targets via KRAB-containing zinc finger proteins and their co-repressor KAP1. MERVL repression is dependent on the presence of H3K9Me2 deposited through the combined action of G9a/GLP **(D)**. Surprisingly, deletion of a variety of other chromatin proteins including LSD1, RYBP, and KAP1 also derepresses MERVL. The interplay between these pathways is presently unknown. LSD1/KAP1 and RYBP might all be recruited trough YY1-family DNA-binding factors as YY1 independently interacts with either KAP1 or RYBP **(D)**.

In mouse ES cells retrotransposon silencing requires the activity of among others Eset or G9A-mediated histone modification machineries, and polycomb repressive complexes (PRC). Polycomb group (PcG) proteins are chromatin modifiers with important functions in cell proliferation, axial development, and X-chromosome inactivation (XCI) (102). In addition, PcG proteins contribute to pluripotency of mouse ES cells and coordinate genetic programs that orchaestrate differentiation of stem cells and cell fate decisions (103). PcG form two main multiprotein complexes termed PRC2 and PRC1 (104). These complexes bring about chromatin and histone modifications and catalyze the trimethylation of histone H3 at lysine 27 (H3K27me3) and the monoubiquitination of lysine 119 on histone H2A (H2AK119Ub1) for PRC2 and PRC1, respectively. A standard model postulates that chromatin-tethered PRC2 modifies histone H3, the resulting H3K27me3 is bound by the CBX component of PRC1, which in turn deposits H2AK119Ub1 (104). The concerted and sequential activities of PRC2 and PRC1 generate an inhibitory chromatin environment, limiting transcription of nearby genes (see Figure 3).

RYBP was identified as a protein that interacts with both RING1 and Yin Yang 1 (YY1) (105). RYBP is a zinc finger protein, which in addition to its association with PcG complexes through RING1 is also a subunit of complexes containing the OCT4/POU5F1 TF essential for pluripotency in ES cells (106, 107). Despite its interaction with Ring1B, genome-wide chromatin binding of RYBP does not overlap well with PRC1 binding (108).

In addition to recruitment of PRC1 via interaction with PRC2 as outlined above, PcG complexes distinct from PRC1 are also recruited to a subset of CpG islands (CGIs) not marked by H3K27Me3 (109). CGIs lack DNA methylation and are associated with most mammalian gene promoters (110). Variant PRC1-related complexes that bind CGIs contain RYBP, and also KDM2B (FBXL10), which specifically recognizes non-methylated DNA in CGIs. KDM2B in turn recruits PRC1, leading to monoubiquitylation of H2AK119 and repression of associated genes. Yet another variant PRC1 complex also contains both RING1B and RYBP in addition to MEL18 (111) and differs from the canonical PRC1 in that the CBX7 subunit is excluded. The presence of CBX7 in these complexes appears to be mutually exclusive with association of the RYBP subunit (see also Figure 3).

Constitutive depletion of *Rybp* in a mouse model causes lethal gastrulation defects and obviates the generation of ES cell lines (112). By contrast, conditional inactivation of *Rybp* allows for the generation of ES cells with apparent normal expression of stem cell markers (108). Although RYBP associates with a subset of H3K27 and H2K119Ub-modified PRC1 target genes, it contributes only moderately to repression of these PRC1-regulated promoters and is dispensable for the association of PRC1 components MEL18 and RING1B to those targets (108). By contrast, in ES cells RYBP is necessary for repression of *MERVL* (but not musD and IAP) and two-cell stage-specific genes (see below). No direct evidence has yet implicated RING1B/PcG as a necessary cofactor in the observed regulation of *MERVL* levels by RYBP. However, in a limited analysis of mouse ES cells deficient in both RING1B and PRC2 (113), IAP, and RLTR33 (and to a lesser extent ORR1A1, RLTR4, and MMVL30) were upregulated, pointing toward a generic role for PcG in regulation of TE.

## Regulation of *MERVL* by YY1-Family Members

Among the genes showing non-uniform expression in ES cell cultures is *Rex1/Zfp42*, a zinc finger protein with a unique expression pattern in ES cells (114, 115). Modest levels of expression are also detected during spermatogenesis and early trophectoderm derivatives and placenta in the mouse (114, 116). *Rex1* is expressed throughout pre-implantation mouse development (117). Although deficiency of Rex1/Zfp42 affects ES cells only weakly (20, 115) expression is frequently used to identify pluripotent stem cells (118). REX1 has an essential role in the initiation of XCI in female ES cells (19), through regulation of the lncRNA *Tsix* (18). Although depletion of *Rex1* does not affect expression levels of pluripotency markers (except for *Tsix*), it allows for upregulation of *MERVL* expression (20). REX1 has been shown to bind to *MERVL loci* in *in vivo* chromatin binding assays. Regulation of *MERVL* by REX1 is even more pronounced *in vivo*, as transient gain-and-loss of REX1 both influence *MERVL* levels during the late cleavage stages of the embryo, in a way compatible with a silencing function for REX1 (20). Thus, REX1 contributes to silencing of ERV expression in mouse ES cells and during pre-implantation development.

REX1 shares high homology in the DNA-binding zinc fingers with the well-studied, ubiquitously expressed TF YY1. YY1 is the prototype of a small family of DNA-binding TFs that also includes YY2 (119). Dependent on the epigenetic environment and numerous interactions with histone modifying complexes, YY1 can function as an activator, repressor, or initiator of gene transcription. YY1 contributes to the control of imprinted genes (120) and genes encoding regulators of the cell cycle (121), especially cytokinesis. Intriguingly, YY1 has been described already in the early 90s as a factor bound upstream of IAP and MMLV elements (122, 123). In addition, silencing of MMLV in embryonic cells is also dependent on YY1 (91). In line with the high homology in the DNA-binding zinc fingers between REX1, YY1, and YY2 (119), the latter two also bind both IAP and muERV-L elements, and weakly to musD in ES cells [Ref. (20); Raquel Pérez-Palacios, Pedro Muniesa, and Jon Schoorlemmer, manuscript in preparation]. In contrast to more efficient binding of REX1 in ES cells to muERV-L, YY1 and YY2 preferred IAP (20).

Consistent with *in vivo* chromatin binding, YY1-family proteins also play a role in regulation of a distinct subset of ERVs. While selected ERV class I and II elements are silenced by YY1 (in F9 EC cells), class III elements including muERV-L are not (91). Repression of IAP is also dependent on KAP1 in mouse ES cells and in early embryos (124). As KAP1 binding to IAP LTRs is lost in YY1 KD cells (91), and YY1 directly interacts with both KAP1 (91) and RYBP (105), YY1 may bridge RYBP/PRC1 and KAP1-dependent silencing (Figure 3). By contrast, REX1 (in mouse ES cells) contributes to silencing of muERV-L and musD (Class II) to a minor degree (20). Combined, these results are compatible with an important role for YY1-family members in ERV silencing.

Although RYBP interacts *in vitro* with YY1 (105) as well as with the related YY2 and REX1 proteins (125), neither of these proteins form part of RYBP-containing PcG complexes (111). As YY1-binding sites in ES do not significantly overlap with PRC1 nor with PRC2 binding (126), it was postulated that YY1 is unlikely to be relevant for PcG-dependent gene regulation (111, 127). However, this conclusion was reached ensuing the analysis of canonical protein-encoding target genes. In view of the data showing muERV-L deregulation in *Rybp*-deficient ES cells, we suggest that REX1 (and/or YY1 and YY2) may recruit RYBP and associated PcG/PRC1 complexes to binding sites in retroviral elements, ultimately contributing to silencing. This hypothetical model is depicted in Figure 3.

## Different ERV Classes are Regulated by Distinct Epigenetic Mechanisms in Mouse ES Cells

Histone H3 lysine 9 methylation (H3K9me2/3) was initially observed as modification associated with centromeric repeat DNA and therefore presumed to be involved in repression (128, 129). It was subsequently observed that silencing of the promoter of the *Oct4/Pou5f1* gene during differentiation is associated with the presence of H3K9me3 (130). In ES cells, different ERVs also carry this mark.

In accordance with H3K9me3 and H4K20me3 marking of ERVs specifically in ES cells (131), a role for SETDB1 in regulation was postulated. Indeed, a wide variety of Class I and II ERVs including ERVK10C and other IAP elements, musD, and ETn elements have lost the H3K9me3 mark and are ectopically expressed in *Setbd1*-deficient cells (100, 101). SETDB1 (also known as ESET) is recruited to chromatin through interactions with the KAP1 co-repressor (132). KRAB-associated protein 1 (KAP1) is a co-repressor of transcriptional repressors of the Zinc Finger Protein with Krüppel-Associated Box (KRAB-ZFP) family (133).

KRAB-associated protein 1 (also known as TRIM28 or TIF1β) recruits HDAC and histone methyltransferase machinery to its chromatin targets. *Kap1* deficiency causes developmental arrest shortly after implantation and gastrulation failure (134). *Kap1* cooperates with *Cnot3, c-Myc*, and *Zfx* in functions essential for the maintenance of pluripotency and self-renewal of ES cells (107, 135). Following up on initial reports that KAP1 mediates silencing of an exogenous virus in ES cells (136, 137), it was subsequently discovered that KAP1 is essential for repression of a large set of endogenous retroelements (100, 124). KAP1/SETDB1 complexes are tethered in turn to TE targets through interactions with KRAB-ZFP.

The genomes of higher vertebrates (including mice and humans) encode a large group of over 300 TFs named KRAB-containing zinc finger proteins (138). They contain a varying number of C2H2 zinc fingers (ZF) that bind DNA in a sequence-specific way and an N-terminal Krüppel-associated box (KRAB) domain with transcriptional repression activity (133). As the KRAB domain associates with KAP1, it was proposed that the sequence-specific docking of KRAB-ZFPs at given genomic *loci* can induce transcriptional repression mediated by KAP1 and associated proteins (139). This silencing mechanism is important in ES cells and operates during preimplantation development (90), in accordance with high expression of *Kap1* at this stage (Figure 2).

More recent results indicate that different classes of ERV are regulated by distinct chromatin-modification pathways. Class III *muERV-L/MERVL* and MaLR (i.e., ORR1A3) families are generally devoid of the H3K9me3 mark in ES cells (101, 131). By contrast, *MERVL* elements carry the H3K9me2 mark, and de-repression is associated with loss of H3K9me2 (77). Moreover, while repression of both ERV I and ERV II elements in ES cells is dependent on SETDB1, this is hardly the case for ERV III (60, 100, 101). *MERVL* silencing is dependent on chromatin association and HMTase activity of both G9a (EHMT2) and GLP (EuHMTase1), two closely related HKMT that form a heteromeric complex *in vivo* (60). The complex represents the main HKMTs to catalyze H3K9me1 and H3K9me2 in euchromatin. Deletion of either G9a or GLP in ES cells dramatically reduces overall H3K9me1 and H3K9me2 levels (140, 141). The mechanisms underlying G9a recruitment to genomic targets including *MERVL* are not understood. G9A/GLP does not interact with either KAP1 or KRAB-ZFP (142). This observation is consistent with the notion that a wave of *MERVL* expansion has taken place in the mouse relatively recently (32), potentially precluding KRAB-ZFP adaptation to these novel sites. Recruitment mechanisms may rely on other chromatin proteins that reportedly interact with G9A/GLP, including Blimp1, ZNF200, HP1, and DNMT1 (142).

However, class III including *MERVL* ERVs are also upregulated in ES cells following *Kap1* depletion (124). Silencing relies on targeting through the 5′UTR/LTR (124). *MERVL* expression is also de-repressed in ES cells that lack LSD1/KDM1 (87), a mono- and di-methyl lysine-specific histone demethylase 1 (77). Consistent with the detection of histone modification changes in *Lsd1*-mutant ES cells that are incompatible with LSD1 specificity, additional modifiers copurify with LSD1 complexes including KAP1-associated HDAC. HDAC acts in concert with LSD1 to properly repress muERV-L/*MERVL* in pluripotent stem cells (77, 143).

*MERVL* repression is dependent on the presence of H3K9Me2 deposited through the combined action of G9a/GLP (60) and LSD1/HDAC1 activity, although the interplay between these two pathways is presently unknown. Furthermore, although RYBP is not a component of LSD1 complexes (77), this PcG-associated protein also plays a role (see Figures 3B,D). As outlined above, RYBP may contribute through association with either REX1 or YY1. The former may attract demethylating activity through its interaction with LSD1 (20), while YY1 was shown in turn to selectively bind KAP1 in undifferentiated stem cells (91), providing a potential docking site for KAP1 and its associated activities.

The variety of potentially independent mechanisms contributing to *MERVL* silencing could be explained by two different but not mutually exclusive arguments. First of all, different copies of *MERVL* may be regulated by distinct mechanisms. The mouse genome contains 500–600 full-length and nearly 350 protein-coding *MERVL* elements (60, 61). Except the LSD1-deficiency, which affects many different copies (77), in most studies describing de-repression of *MERVL* the difference between induction of multiple copies versus several fold induction of a few copies has not been addressed. Alternatively, different mechanisms may be initiated at different time-points of the life cycle, which is discussed below.

## The Contribution of Retrotransposons to Gene Expression Specific for Totipotency and Pluripotency

In contrast to silencing of retrotransposons in differentiated cells and tissues, activity of retrotransposons in the germline and/or in pluripotent cells destined for the next generation is a crucial prerequisite to ensure genetic propagation. In line with the resulting restricted and regulated expression patterns, retrotransposon-derived control elements underlie transcriptional networks in pluripotent stem cells. The binding regions for several core pluripotency factors including SOX2, NANOG, and POU5F1 show meaningful overlap with degenerated TEs, i.e., ERV-derived LTRs. In particular, remnants of class II elements (IAP and ETn) overlap binding sites for SOX2/NANOG/OCT4 in the mouse (49, 144). Despite evolutionary innovation and diversification at the level of individual binding sites, overlap with retrotransposons is evident in both mouse and human (49). Hence, ERV-contributed cis-binding sites may underlie the coordination of gene expression in pluripotent cells in a species-specific manner. The prevalence of MERVL-derived control elements and LTRs (Class III ERV elements) in the vicinity of two-cell stage gene promoters (17, 60), suggests that they orchestrate coordinated regulation of gene expression at this stage. The contribution of ERV-derived sequences to cis-acting regulatory elements is not restricted to mammals (47, 48, 144). A chicken retroviral (Ens-1) LTR contributes to both the transcriptional networks in ES cells and to extraembryonic transcription (145).

In placental mammals (Eutherians), female cells silence one of their X-chromosomes in a process called XCI to accomplish equal expression levels of X-encoded genes compared with males (146, 147). In the mouse, the activity of both X chromosomes is re-established in pluripotent cells in the ICM. This process calls for the expression of the lncRNA *Tsix*. The activity of both X-chromosomes in female ES cell lines is similarly dependent on *Tsix*, which counteracts painting of one of the X-chromosomes by *Xist*. The level of *Tsix* RNA depends, in addition to the promoter, on a cis-acting element called DxPas34 (148). In rodents DxPas34 contains binding motifs for several TFs including CTCF and REX1 [Ref. (18); Guallar et al. submitted] and DxPas34 activity is dependent on the presence of REX1 (in addition to c-MYC and KLF4) (18). Surprisingly enough, the DxPas34 element has the structure of a repeat element very similar at the sequence level to ERVs (149). In fact, it is most similar to, and can be considered a fossilized version of muERV-L/MERVL. The activity of this element seems unrelated to LTR-originated promoter activity, as the sequence similarity is in the internal portion of the retroviral element, and not in the LTRs. Although XCI is a female-associated process (while most commonly used mouse ES lines are male), re-activation of both X-chromosomes is a hallmark of pluripotency. Naive female ES cells invariably express *Tsix* resulting in the presence of two active X chromosomes. This observation suggests that MERVL-related sequence elements direct gene expression required for naive pluripotency (75).

Several genes identified initially as transcripts expressed exclusively in two-cell stage embryos represent transcripts that initiate in MERVL/MT2 LTRs. These include Zscan4, Tcstv1/3, Eif1a (Gm2022), similar to Tho4, Tdpoz1–5, and Zfp352 (16, 61, 64, 78, 150). We will refer to these genes as LTR-linked 2C genes. ES cells devoid of individual *MERVL* regulators discussed above all suffer de-repression of both *MERVL* and (a subset of) LTR-linked 2C genes. This holds in addition to LSD1 for G9A (60), KAP1 (101), HDAC1 (77), and RYBP (108). Although de-repression of muERV-L might be a consequence of ectopic expression of LTR-linked 2C genes, no direct evidence for such a mechanism is available. Therefore, we favor the hypothesis that aberrant transcription of 2C genes results from sharing the silencing machinery with MERVL. In line with this hypothesis, *MERVL*-positive ES cells are believed to represent a transient two-cell state accompanied by induction of LTR-linked 2C genes.

## Developmental Potency and Developmental Timing

In contrast to the well-established exclusive contribution of ES cells to the ICM of the blastocyst of chimeras (and embryonic tissues during later development), 2C-like cells also contribute to the trophectoderm and to the yolk sac and placenta in later embryos. The proportion of 2C-like cells within an ES cell culture is controlled by LSD1, and to a lesser extent by LSD1-associated HDAC1 (17). Without LSD1/HDAC1 activity, more cells reside in the 2C-like state. Not surprisingly then, *Lsd1*-deficient ES cells also show enhanced lineage potential in mouse chimera assays and contribute to embryonic tissues and primordial germ cells, the amnion, yolk sac, and placental tissues, including giant trophoblast cells. Therefore, the separation between trophectodermal and embryonic lineages established in post cleavage-stage embryos is overturned in 2C-like cells, which transiently exhibit an expanded cell fate relative to pluripotent cells. In ES cells RYBP is necessary for repression of germline-specific genes, as well as for repression of MERVL class ERVs (not musD norIAP) and two-cell stage-specific genes (including *Tcstv3, Zscan4, Zfp352*, and *Ubtfl1*). In addition, the collection of genes controlled by RYBP overlapped very well with LSD1 target genes (77, 108), including a set of extraembryonic endoderm markers. This coincidence reinforces the hypothetical model presented (in Figure 3) suggesting that RYBP and LSD1 act in concert.

Neither self-renewal properties, nor pluripotency (measured *in vitro* or by contribution to chimeric embryos) have been rigorously established so far for the majority of ES cells devoid of individual *MERVL* regulators discussed above (except for *Lsd1* and *Rybp*). However, available data are compatible with the notion that deficiency in these MERVL regulators causes defects during differentiation (100, 151), which can be interpreted as altered pluripotency. It is not clear at present whether the shared de-repression of *MERVL* in these cells drives a higher percentage of cells into a 2C-like state which in turn allows differentiation into extraembryonic lineages. Alternatively, de-repression of *MERVL* and 2C-stage-specific transcripts initiating on ERV may be indicative of transcriptional deregulation, allowing for enhanced plasticity of cell fate and increased extraembryonic differentiation.

**Figure 4:**
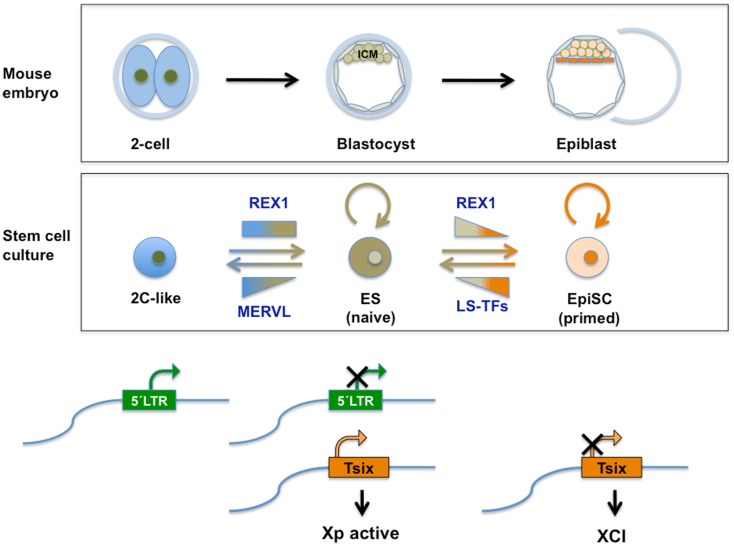
**Top panel: schematic representation of relevant stages during mouse pre-implantation development**. A specific set of genes transiently expressed at the two-cell mouse embryo (see also Figure 2), contributes to the totipotent status distinctive of those early stages. See text for further information. Middle panel: transient subpopulations of mouse ES cells. MERVL-derived elements drive the expression of genes specific to two-cell embryos in a transient subset of ES cells, discriminating pluripotent ES cells with ICM characteristics from totipotent 2C-like cells with expanded developmental potential. Independently, self-renewing (OCT4/SOX2 positive) ES cells shuttle between a so-called naive pluripotent status of gene expression and a primed state, characterized by the ability of the former to contribute to germline chimeras. These populations can be separated based on the expression of various heterogeneously expressed genes including *Rex1*. Among other characteristics of naivety, figures high expression of *Rex1* in all cells, as opposed to heterogeneous or low expression. Primed cells exhibit a lower potency of development and no expression of *Rex1*. These populations bear similarity to ICM cells in the blastocyst and Epiblast Stem cells (EpiSC), respectively. REX1 contributes to regulation of MERVL-derived transcriptional control elements relevant to these cellular transitions. REX1 is present throughout pre-implantation development (Figure 2) and regulates *MERVL* and *Zscan4* expression at these stages. Therefore a function of REX1 in the transition between totipotent 2C-like and pluripotent ES cells is speculated on (see text). Levels of *Zfp42* are not significantly affected in 2C-like cells (17). On the other hand, REX1 positively affects re-activation of the inactive X-chromosome typical of the ICM and naive ES cells, by promoting the transcription of Tsix. Bottom panel: LTR regulation. The LTR drives expression of *MERVL* and totipotency-specific gene expression in 2C-like cells with totipotent characteristics, which co-exist in culture with pluripotent REX1-expressing ES cells. REX1 silences *MERVL* in ES cells, and is necessary for full expression of *Tsix*. *Tsix* expression in turn is instrumental in both the re-activation of the paternal X-chromosome in the blastocyst, which is silenced at earlier stages and maintenance of re-activation compatible with naive pluripotency. In primed ES cells and in EpiSC *Tsix* is silenced and XCI is installed. LS-TFs, lineage specific transcription factors expressed in primed ES cells and EpiSC; Xp, paternally derived X-chromosome XCI; X-chromosome inactivation; Tsix, lncRNA counteracting XCI, whose transcription is driven by a MERVL-related repeat sequence.

The absence of REX1 in mouse ES cells is accompanied by a (relatively minor) de-repression of *MERVL*, suggesting that REX1 may control only few copies among the *MERVL* elements (20). It is unclear at present whether this induction reflects increased *MERVL* expression in a constant number of expressing cells, or results from an increased subpopulation of 2C-like cells. In the latter case, REX1 may negatively influence transition into the 2C-like state, or stimulate reversal into pluripotent cells (Figure 4). The latter would be in accordance with its repressing function on *MERVL* after its peak expression in two-cell embryos (20). REX1 may regulate this transition indirectly through its interaction with LSD1 (20), or directly as a transcriptional repressor in concert with RYBP (125). A final alternative resides in a distinct function of REX1 in protecting a regulatory ERV *locus* from generic TE silencing mechanisms. The above considerations imply that REX1 is present in 2C-like ES cells based on the comparison with two-cell embryos. In support of this view, *Rex1* mRNA levels were not identified as differentially regulated between 2C-like and pluripotent ES cells (17). REX1 is subject to RNF12-mediated ubiquitination and subsequent degradation in pluripotent cells (19). It remains possible that a similar mechanism lowers REX1 protein levels in 2C-like cells.

A potential enhanced contribution of *Rex1*-deficient cells to extraembryonic tissues in chimeras has not been rigorously tested [see Ref. (115)]. The influence of *Rex1* on developmental potency *in vitro* concerns some extraembryonic endoderm markers, but is much more restricted than reported for *Lsd1* and *Rybp*. A role for *Rex1* in *MERVL*-driven totipotency is not easily reconciled with the reported differentiation characteristics of *Rex1*-deficient ES cells *in vitro* (115), potentially due to the separate role of *Rex1* in naive pluripotency (Figure 4).

Based on the different requirements for RYBP during the generation of ES cells from blastocyst versus depletion after lines have been established, it has been suggested that *Rybp i*s required for the generation, but not for the maintenance of the ES cells state (108). This notion coincides with the magnitude of *MERVL* regulation by *Rex1* during these stages. It is therefore a possibility that REX1 attracts a repressing complex to *MERVL* during pre-implantation development via its interaction with RYBP, while repression at later stages (including in the ICM/ES cells) is exerted in conjunction with parallel pathways.

Compared to two-cell embryos, an increase in H3K9me2 methylation at heterochromatin has been reported at the four-cell stage (152), with the increased level maintained up to the blastocyst stage. A similar increase associated with *MERVL* elements may underlie G9A-mediated repression of *MERVL* after transient expression in two-cell embryos.

## Zscan4, Pluripotency, and Two-Cell Embryos

An alternative pathway conferring characteristics of two-cell embryos on ES cells implicates the *Zscan4 locus*. *Zscan4* is the common name for several transcripts generated from a cluster of six paralogous genes with high homology (73). ZSCAN4 is a nuclear protein with DNA-binding Zinc Fingers and a SCAN domain for protein–protein interactions. In a pattern reminiscent of *MERVL, Zscan4* is expressed exclusively in two-cell embryos and transiently and reversibly in only a small percentage of ES cells in culture (73, 153). *Zscan4*-expressing cells selected to near homogeneity (using a reporter gene inserted in the *locus*) do exhibit standard pluripotency characteristics such as expression of OCT4/POU5F1, *in vitro* differentiation into three germ layers and contribution to chimeric embryos. Despite the relatively small percentage of Zscan4-expressing cells in an ES culture at a given time point, all cells cycle through a Zscan+ state over a span of 9–10 passages. Failure to do so results in cell death associated with telomerase-independent telomere shortening, loss of normal karyotype, and genomic instability. These observations are consistent with a role for ZSCAN4 in the lengthening of telomeres through a recombination process unique to one- and two-cell embryos called sister-chromatid exchange (154).

At this moment, there is no direct proof that implicates ZSCAN4 in regulation of either *MERVL-*driven transcription or *MERVL*-directed passage to the 2C-like state (17). They seem to be coregulated as *Zscan4* is manifold induced in 2C-like cells, depletion of 2C-like cells also depleted *Zscan4* levels (17), and both *Zscan4* and *MERVL* are upregulated in KAP1- and LSD1-deficient ES cells, and to a minor extent in G9A deficient cells. Hence, ZSCAN4 might directly regulate *MERVL*-driven conversion to 2C, and/or *MERVL* may somehow coordinate induction of genes required for SCE. Integration of both processes might rely on REX1, which has been shown to regulate expression of *MERVL* in both early mouse embryos and ES cells (20), and *Zscan4* in embryos (117). A difference between regulation of *Zscan4* and *MERVL* expression is the dependence of *Zscan4* levels on the presence of REX1 in two-cell stage embryos (117). By contrast, *MERVL* expression is indifferent to REX1 levels at this stage (20), suggesting a potential mechanism underlying the differences between *Zscan4*- and *MERVL*-expressing ES populations.

## Human ERV and Embryonic Stem Cells

The contributions of *MERVL* to gene expression restricted to different states of developmental potency, and its epigenetic control mechanisms (Figures 3 and 4) have been described in mouse stem cells. Studies to determine the role of similar *HERV* -driven processes for HESCs are ongoing. We will briefly discuss present knowledge and issues being addressed in this respect. Definitely, many differences exist between HESC and mouse ES cells (155). Although pluripotent, the differentiation properties and growth factor dependency of HESC are more similar to mouse Epiblast Stem Cells [reviewed in Ref. (14)]. If remnants of *MERVL*-related sequences are present in human they are much more degenerated (156). Whether human MERVL-related sequences that influence TSIX expression and XCI in HESC exist, is an unresolved issue at present (157, 158). Surprisingly, few data are available regarding the expression of HERV in human tissue in general. In HESC, HERV-K expression is very high (159). As opposed to a wide variety of differentiated cells, this element is marked in HESC with the activity associated H3K4Me3 modification. Moreover, binding sites for the core pluripotency factors NANOG, OCT4, and SOX2 are located within HERV-K LTRs. HERV-K(HML-2) RNA and protein are both expressed in undifferentiated HESCs (160). As exemplified by rapid silencing upon differentiation, HERV-K expression is representative of the pluripotent state of HESC (159, 160), potentially displaying regulation similar to *MERVL* in the mouse. HERV hardly displays retrotransposition activity, as opposed to rodent ERVs. However, present inactivity does not rule out its previous recruitment as cis-control element. Considering the dearth of information on gene expression in human embryos and placenta (161, 162), HERV expression may be more prominent than presently known. By contrast, as a result of expanded life span, human cells are equipped with a more extended set of anti-tumor mechanisms compared to rodents. It cannot be excluded that expanded life span also has required a tighter control of TEs, which has evolved differently from rodents.

Moreover, the *muERV-L/MERVL* regulator REX1 is a well-defined marker for both mouse and HESCs (163, 164). REX1 expression also defines heterogeneity within OCT4 and TRA-1-60 positive populations of HESCs (164). Low or absent REX1 expression identifies an OCT4 positive cell type that does not revert back to REX1 positivity under normal conditions and is prone to differentiation into definitive and extraembryonic endoderm. It is an outstanding question whether pluripotency in HESC is fine-tuned by REX1-mediated regulation of HERV-K similar to the situation in the mouse.

In human pluripotent cells, cellular heterogeneity has been studied mainly in the context of standardization of human induced pluripotent stem (HiPS) cells. HiPS are pluripotent cells with characteristics identical to or very similar to HESC, which can be generated by reprograming of adult cells (14, 165). Different lines of HiPS cells display a higher level of variability at the level of gene expression and epigenetic signatures compared to ES from either mouse or human. This can be attributed to differences in derivation methods including donor cell types, and to the differences in age, genetic background, and pathology states of the donors (14). However, overcoming longstanding impediments, HESC with properties similar to ground state pluripotency described in the mouse have recently been derived (166). Conditions to generate such ground state HESC/HiPS cells are expected to diminish the cellular heterogeneity observed at the gene expression level. As HERV-K expression is very high in HESC (159), it will be of interest to see if ERV-driven reporters could now be used to classify HESC according to potency levels similar to mouse ES cells.

An important difference between mouse and HESCs also is the latter’s capacity for trophectodermal differentiation. Placentation is a relatively recent event on the scale of evolution, and the diversity of placental morphology among mammalian species suggests that it has appeared various times independently (167). Moreover, generic difference in genome management may already underlie different timing of ZGA between mouse and human (161), and posterior separation of lineages. Such species-specific differences may also have altered cis-acting functions and regulation of ERV-L in different species.

## Concluding Remarks: Relationships with Cancer

We finally comment on the relevance of *HERV* -directed transcription and the mechanisms that control it for human cancer biology. Cancerous cells often display genome-wide loss of DNA methylation resulting from a loss of methylation on TE (24, 168), and progressive loss of silencing. Similar to DNA hypomethylation, dramatic changes in histone modification patterns are rampant (169). Relaxation of alternative silencing mechanisms described here may re-activate TE/ERVs elements in tumors, as initially demonstrated in mice (170).

Mechanisms that explain how the activity of HERVs may contribute to tumor formation have been reviewed elsewhere [i.e., Ref. (8, 171)]. Both *de novo* transposition events and deregulation of HERV-derived control elements may affect the expression of tumor suppressor genes or (proto-)oncogenes nearby. Occasionally, proto-oncogenes may be incorporated in variant HERV. As these genes control cell growth and proliferation, their deregulation may give rise to a malignant or transformed cell with enhanced growth characteristics. In addition, HERV-encoded ENV proteins may provide cell fusion properties that help the transition toward metastasis or help tumor cells evade an anti-tumoral immune response by virtue of its immunosuppressive properties (172). Alternatively, uncontrolled expression of TE has been related to genome instability caused by DNA breaks and chromosome translocations. Deregulation of retroviral elements in tumors might be indicative of improper activity of genome stability mechanisms, potentially preceding insult resulting from de-stabilization. RT activity (encoded in HERV) might be used as a measure of the extent of ERV/TE-deregulation (173), and be instrumental in tumor classification. Moreover, as pluripotency markers are overexpressed in tumor cell lines coinciding with increased RT activity (173), the level of RT activity might even indicate the presence of tumor stem cells.

The potential contribution of HERV to tumor formation should also be taken into account in the context of gene therapy, as insertion of new genes in a location that relieves HERV silencing may trigger these activation processes. Moreover, as modified viruses are commonly used as vectors for gene therapy, the relief of HERV silencing commonly associated with viral infections and inflammation (174) constitutes a risk factor for gene therapy by itself and HERV-encoded proteins might provide essential proteins that allow the mobilization of replication-defective provirus.

YY1 overexpression has been reported in tumors, and overexpression levels are related to clinical progression (175). Differences (usually overexpression) of YY1 transcript levels in tumor tissue relative to normal counterparts have also been extracted by computational analysis from gene expression omnibus (GEO) datasets (176). It seems obvious to presume that YY1’s well documented function in the regulation of gene networks that control cell proliferation underlies its role in oncogenesis. However, YY1 participates in distinct mechanisms potentially related to oncogenesis, including increased mutagenesis, regulation of chromosome dynamics (such as imprinting and X-inactivation), and DNA repair and chromosome segregation (177). We suggest that the contribution of YY1-family proteins to oncogenesis may as well reside in silencing of TE (and ERV in particular).

The judge is still out on the question whether HERV activation is a triggering event in tumor development, or whether they are upregulated as a consequence of previous alterations associated with tumor development, i.e., genomic instability or demethylation. So far, the expression of HERVs has been linked to germ cell tumors, breast cancer, ovarian cancer, and melanoma [reviewed in Ref. (8, 10)]. Enhanced knowledge of the mechanisms that control HERV expression will aid the understanding of how heterogeneous HERV activity levels may contribute to differences among individuals in susceptibility to cancer.

## Conflict of Interest Statement

The authors declare that the research was conducted in the absence of any commercial or financial relationships that could be construed as a potential conflict of interest.
